# Saliva Sample‐Based Non‐Invasive Carrier Screening for Spinal Muscular Atrophy, Hereditary Hearing Loss, and Thalassemia in 13,926 Women of Reproductive Age From South Zhejiang

**DOI:** 10.1002/mgg3.70064

**Published:** 2025-02-23

**Authors:** Chenyang Xu, Yanbao Xiang, Xiaoling Lin, Qifan Ma, Yunzhi Xu, Huanzheng Li, Shaohua Tang, Xueqin Xu

**Affiliations:** ^1^ Key Laboratory of Birth Defects, Department of Genetics Wenzhou Central Hospital Wenzhou China

**Keywords:** carrier screening, hereditary hearing loss, SMA, thalassemia

## Abstract

**Background:**

Although spinal muscular atrophy (SMA), hereditary hearing loss (HL), and thalassemia are common monogenic genetic diseases, the carrier frequencies and variant spectrums of these diseases show regional differences, even within China. Their carrier frequencies and variant spectrums in Southern Zhejiang, China are unclear.

**Methods:**

Saliva was collected for carrier screening and amniotic fluid, villi, and peripheral blood were collected for prenatal diagnosis. Real‐time quantitative polymerase chain reaction (PCR) and multiplex ligation‐dependent probe amplification (MLPA) were used to detect the copy number of *SMN1* exon 7. PCR coupled with flow‐through hybridization, MLPA, and Sanger sequencing were used to detect common genes for HL and thalassemia.

**Results:**

Common variants were detected in 15.14% (2109/13926) of the 13,926 women of reproductive age from South Zhejiang who participated in this study. The carrier frequencies of SMA, HL, and thalassemia were 2.11% (294/13926), 4.87% (678/13926), and 8.82% (1228/13926), respectively. In total, 56.47% (1117/1978) of husbands were successfully recalled. The total number of at‐risk couples was 111 (111/13926, 0.80%). Further, 47 families underwent prenatal diagnosis. A total of 13 (13/13926; 0.93‰) affected pregnancies were identified.

**Conclusion:**

Our findings confirm that SMA, HL, and thalassemia are highly prevalent in Southern Zhejiang, with some regional specificity, as compared with recent large population‐based surveys in China. Further, a rapid saliva sample‐based non‐invasive screening method was established, and its feasibility was demonstrated.

## Graphical abstract

We established a rapid saliva‐based non‐invasive screening model for carriers of SMA, hearing loss, and thalassemia among women from South Zhejiang and provided prenatal diagnosis for high‐risk families. The results not only clarified the local carrier frequency of these diseases but also confirmed the feasibility of the carrier screening model.

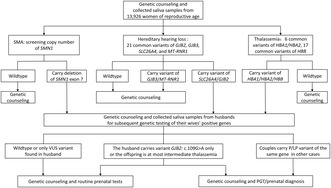

## 1 Introduction

At present, more than 8000 monogenic genetic diseases have been identified; these diseases are among the important causes of birth defects in newborns. Monogenic genetic diseases are detected in about 1% of newborns, accounting for 10% of infant mortalities and 20% of pediatric hospitalizations (Kumar et al. 2001; Wojcik et al. 2019). Most monogenic genetic diseases lack effective treatments; however, carrier screening combined with genetic counseling and prenatal diagnosis can increase reproductive autonomy for at‐risk couples. The American College of Medical Genetics (ACMG) recommends that information on carrier screening, for at least the most common inherited genetic conditions, should be offered during pregnancy and preconception (Gregg et al. 2021).

Spinal muscular atrophy (SMA), hereditary hearing loss (HL), and thalassemia are three common monogenic hereditary diseases. Major thalassemia and SMA are important causes of birth defects. In contrast, HL is not considered a major birth defect in many countries, and many different treatments for HL available. However, it does pose economic and psychological burdens for affected families. Carrier screening for these diseases is recommended by various guidelines and expert consensus. More than 95% of SMA cases are caused by homozygous deletion of survival of motor neuron 1 (*SMN1*; OMIM 600354) exon 7 with an incidence of approximately 1:10,000. HL affects 1‰–2‰ of newborns, and more than half of these cases have genetic causes (Li et al. 2022; Morton and Nance 2006). The main genes implicated in HL among the Chinese population include gap junction protein beta 2 (*GJB2*; OMIM 121011), gap junction protein beta 3 (*GJB3*; OMIM 603324), solute carrier family 26 member 4 (*SLC26A4*; OMIM 605646), and mitochondrially encoded 12S rRNA (*MT‐RNR1*; OMIM 561000) (Zhang et al. 2023). Thalassemia is widely distributed in the tropics and subtropics. Most cases are caused by variants of hemoglobin subunit alpha 1 (*HBA1*; OMIM 141800), hemoglobin subunit alpha 2 (*HBA2*; OMIM 141850), and hemoglobin subunit beta (*HBB*; OMIM 141900) (Weatherall 2005).

There are no centralized screening data for SMA, HL, and thalassemia among reproductive‐aged Chinese women, and previous reports indicate that the spectrum of variants associated with these diseases varies across different regions in China. The reported carrier frequencies of SMA range from 0.98% to 2.02% among different populations (Sugarman et al. 2012), and the frequencies in various regions of China range from 1% to 2.11%. These estimates are generally lower than those of Poland, Saudi Arabia, and India (Zhou et al. 2024). The frequency of HL‐associated variants among Chinese newborns was found to be 5.12%, which is substantially different to the rates in other ethnic populations (Zhang et al. 2023). Thalassemia is widely prevalent in Mediterranean coastal areas, Southeast Asia, and the Middle East. Currently, the available reports on carrier frequencies in China have mainly focused on southern China, including Guangdong, Guangxi, Sichuan, Yunnan, Guizhou, and Fujian Province, with obvious regional differences detected, ranging from 6.81% to 19.49% (Du et al. 2024; Huang et al. 2019; Xian et al. 2022; Yang et al. 2023; Yin et al. 2014). Zhejiang Southern Region is located in the southeastern region of China, with a permanent population of over 10 million. At present, there is a lack of statistical data on the carrier frequencies and spectrum of variants of these diseases in our region. It is necessary to investigate the local molecular epidemiological characteristics of these diseases to help asymptomatic carriers of related diseases in this region to better understand their reproductive risks and options.

Factors such as significant population mobility, inconvenient medical treatment, regional screening implementation, and a lack of public awareness have brought about great challenges in carrier screening. It is known that the oral mucosa cells in saliva contain a complete human genome DNA sequence, and a standard saliva sample can be used for effective genetic testing. Here, we established a non‐invasive screening method based on saliva genetic testing to mitigate the problems mentioned above. Using this screening method, an epidemiological investigation and variant spectrum analysis of common genes for SMA, HL, and thalassemia were conducted in women of reproductive age in South Zhejiang, China.

## 2 Materials and Methods

### 2.1 Ethical Compliance

This study was approved by the institutional research ethics committee of our unit (No. L2024‐02‐018). The study protocol conformed to the ethical guidelines of the Declaration of Helsinki. Written informed consent was obtained from all participants.

### 2.2 Subjects

**FIGURE 1:**
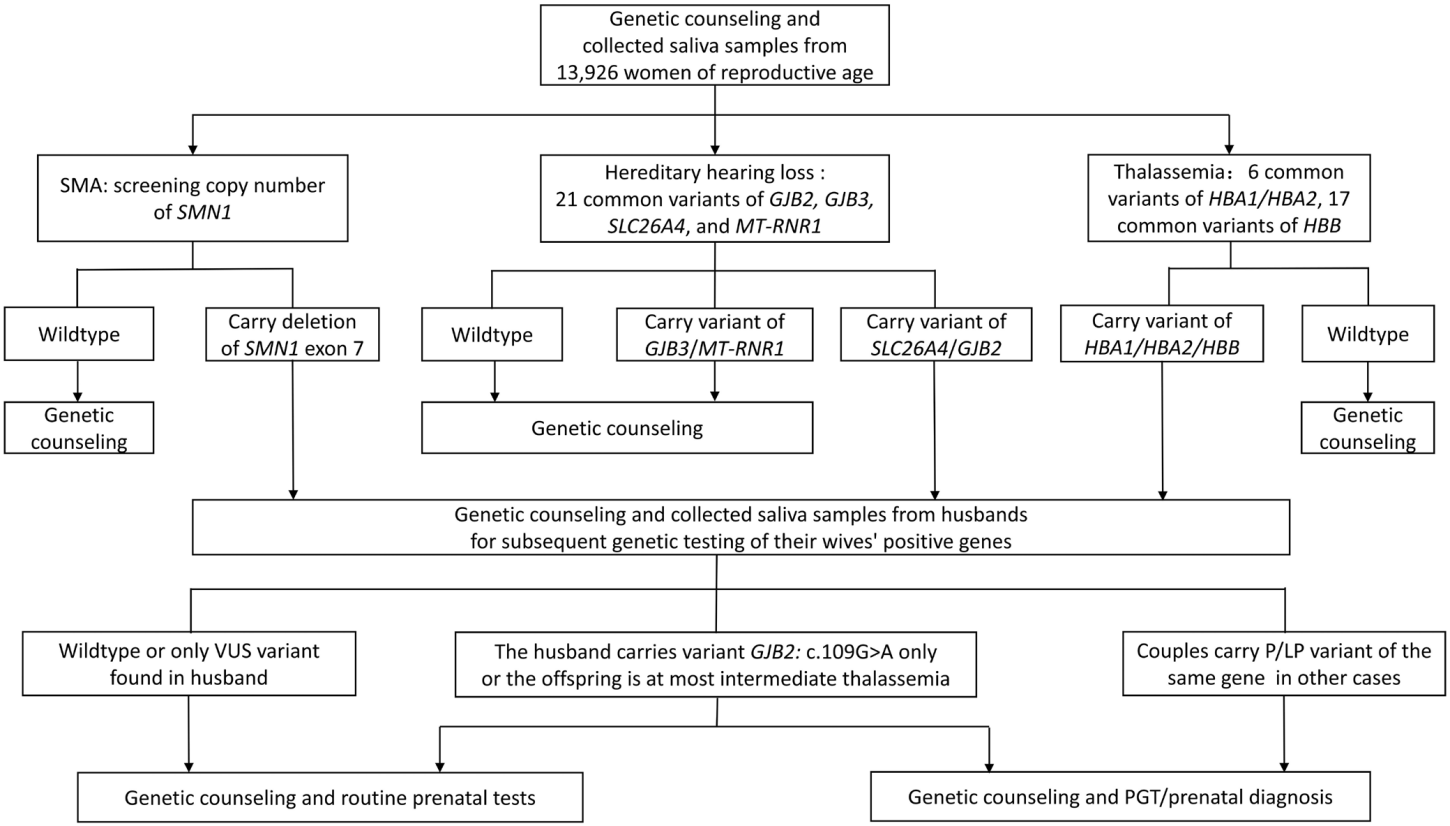
The sequential saliva sample‐based non‐invasive screening method performed for participating females and their husbands.

A total of 13,926 healthy women of reproductive age from South Zhejiang, China, were enrolled in this study between May 2020 and May 2023. They underwent screening for the copy number of the *SMN1* gene (NM_000344.4), six common variants of the *HBA1* gene (NM_000558.5) or *HBA2* gene (NM_000517.6), 17 common variants of *HBB* (NM_000518.5), and 21 common variants of four HL genes *GJB2* (NM_004004.6), *GJB3* (NM_024009.3), *SLC26A4* (NM_000441.2), and *MT‐RNR1* (NC_012920.1). Further, 1117 male spouses were successfully recalled for subsequent genetic testing for their wives' positive genes; *GJB3* and *MT‐RNR1* were excluded from these subsequent analyses. At‐risk couples were offered genetic counseling and prenatal diagnosis at our hospital. A sequential screening method was performed for these couples (Figure 1).

### 2.3 Specimen Collection

Saliva was collected from the female subjects and the participating husbands according to standard instructions. The samples were delivered via courier logistics at 2°C–8°C to the clinical laboratory department for immediate detection or were stored at 2°C–8°C. The peripheral blood of couples undergoing prenatal diagnosis was collected for verification, and fetal samples were obtained by amniocentesis or choriocentesis at the corresponding gestational age. Corresponding reagent strips (Kaishuo Biotechnology Co., LTD., Xiamen) were used to extract genomic nucleic acid from the saliva (C1003 model), peripheral blood (C1001 model), amniotic fluid (C1007 model), and villus (C1003 model) samples. The procedures, including pretreatment, lysis, washing, and elution, were performed with an HF16 plus automatic nucleic acid extractor (Kaishuo Biotechnology Co. Ltd., Xiamen).

### 2.4 SMA Detection

Real‐time quantitative polymerase chain reaction (qPCR) was used to detect the copy number of exon 7 and exon 8 of the *SMN1* gene among female subjects and their participating husbands using an ABI StepOne plus real‐time PCR system (Thermo Fisher Scientific, Carlsbad, CA), according to the manufacturer's protocol (Chromysky Medical Research, Shanghai, China). ΔΔCt > 0.8 was taken to indicate homozygous deletion of exon 7, ΔΔCt > 1.5 indicated homozygous deletion of exon 8, ΔΔCt > −0.45 but < 0.45 indicated heterozygous deletion of exon 7 or 8, and ΔΔCt < −0.55 indicated no deletion. Both multiplex ligation‐dependent probe amplification (MLPA) (P060, MRC‐Holland) and qPCR were performed on samples from families requiring prenatal diagnosis.

### 2.5 Thalassemia Detection

Common variant sites in the *HBA1*, *HBA2*, and *HBB* genes were detected by PCR coupled with rapid flow‐through hybridization (PCR‐FH) technology (Kaipu Biotechnology Co. Ltd., Guangdong) for female subjects and their participating husbands. Six variants associated with the *HBA1*/*HBA2* gene, including three deletion variants ‐^SEA^, ‐α^3.7^, and ‐α^4.2^, as well as three non‐deletion variants, *HBA2*:c.427T>C (α^CS^α), *HBA2*:c.377T>C (α^QS^α), and *HBA2*:c.369C>G (α^WS^α), were investigated. Seventeen variants associated with the *HBB* gene, including c.‐78A>G (−28 (A>G)), c.‐79A>G(−29 (A>G)), c.‐80T>C(−30 (T>C)), c.2T>G (Initiation codon (ATG>AGG)), c.45_46insG (codons 14/15 (+G)), c.52A>T (codon 17 (AAG>TAG)), c.79G>A(codon 26 (GAG>AAG)), c.84_85insC (codons 27/28 (+C)), c.94delC (codon 31 (–C)), c.126_129delCTTT (codons 41/42 (–CTTT)), c.130G>T (codon 43 (GAG>TAG)), c.216_217insA (codons 71/72 (+A)), c.‐11_‐8delAAAC (5′UTR; +43 to +40 (‐AAAC)), c.92+1G>A/T (IVS‐I‐1 (G>A/T)), c.92+5G>C (IVS‐I‐5 (G>C)), c.316‐197C>T (IVS‐II‐654 (C>T)), and c.‐82C>A (−32 (C>A)), were investigated. The experimental procedures and interpretation of results were based on the kit instructions. MLPA (P140, MRC‐Holland) or Sanger sequencing was performed for validation of the samples from families requiring prenatal diagnosis, in addition to PCR‐FH.

### 2.6 Hereditary HL Detection

Twenty‐one common variants in four deafness genes were detected among the female subjects using a HL susceptibility gene kit (Kaipu Biotechnology Co. Ltd., Guangdong) by PCR coupled with rapid flow‐through hybridization (PCR‐FH) technology. These variants included c.508_511dupAACG, c.35delG, c.167delT, c.176_191del, c.235delC, and c.299_300delAT of *GJB2*, c.538C>T and c.547G>A of *GJB3*, c.281C>T, c.589G>A, c.1174A>T, c.1226G>A, c.1229C>T, c.1975G>C, c.2027T>A, c.2162C>T, c.2168A>G, c.919‐2A>G (IVS7‐2A>G), and c.1707+5G>A (IVS15+5G>A) of *SLC26A4*, and m.1494C>T and m.1555A>G of *MT‐RNR1*. The experimental procedures and interpretation of the results were based on the kit instructions. Sanger sequencing was used to determine the sequences of all exons of *GJB2* or *SLC26A4* in the husbands when variants of these two genes were detected in the female subjects. Clinical classification was performed for all detected variants according to the ACMG guidelines (Richards et al. 2015). Both Sanger sequencing and PCR‐FH were performed for samples from families requiring prenatal diagnosis.

## 3 Results

### 3.1 Genetic Carrier Screening of 13,926 Female Subjects

A total of 2246 variants were detected in 2109 (15.14%, 2109/13926) female subjects (Table 1), among which, three subjects (0.14%,3/2109) carried common variants in the genes for all of the investigated diseases (SMA, HL, and thalassemia), 85 (4.03%, 85/2109) carried common variants in genes related to two of these diseases, and 2021 (95.83%, 2021/2109) carried variants in genes related to one of these diseases. The carrier frequencies for SMA, HL, and thalassemia were 2.11% (294/13926), 4.87% (678/13926), and 8.82% (1228/13926), respectively. Among the 2109 carriers, three different variants were found in six cases (0.28%, 6/2109), two different variants were found in 125 cases (5.93%, 125/2109), and a single variant was found in 1978 cases (93.79%, 1978/2109). It should be noted that compound heterozygous variants of the same gene and homozygous variants were also detected in 18 healthy cases (Table 2).

**TABLE 1:** The carrier frequency of common variants in 13,926 women of reproductive age.

Variant	Cases	%	Variant	Cases	%
***SMN1***			***GJB2***		
Exon7 deletion	294	2.11	c.235delC	310	2.23
**SMA Total**	**294**	**2.11**	c.299_300delAT	32	0.23
			c.508_511dupAACG	19	0.14
***HBA1/HBA2***			c.176_191del	18	0.13
‐^SEA^	419	3.01	c.35delG	0	0
‐α^3.7^	279	2.00	c.167delT	0	0
‐α^4.2^	39	0.28	*GJB2* Total	379	2.72
*HBA2*:c.427T>C	22	0.16	***SLC26A4***		
*HBA2*:c.377T>C	16	0.11	c.919‐2A>G	106	0.76
*HBA2*:c.369C>G	12	0.09	c.1229C>T	16	0.11
***HBA1/HBA2*** Total^a^	776	5.57	c.2168A>G	14	0.10
***HBB***			c.1975G>C	6	0.04
c.126_129delCTTT	156	1.12	c.1707+5G>A	6	0.04
c.52A>T	128	0.92	c.1174A>T	5	0.04
c.316‐197C>T	118	0.85	c.281C>T	2	0.01
c.84_85insC	22	0.16	c.589G>A	2	0.01
c.‐78A>G	20	0.14	c.1226G>A	2	0.01
c.79G>A	16	0.11	c.2162C>T	2	0.01
c.216_217insA	5	0.04	c.2027T>A	1	0.01
c.2T>G	5	0.04	***SLC26A4*** Total	162	1.16
c.92+1G>A/T	4	0.03	***GJB3***		
c.‐11_‐8delAAAC	1	0.01	c.538C>T	43	0.31
c.130G>T	1	0.01	c.547G>A	32	0.23
c.45_46insG	1	0.01	***GJB3*** Total	75	0.54
c.‐79A>G	0	0	***MT‐RNR1***		
c.‐80T>C	0	0	m.1555A>G^c^	63	0.45
c.‐82C>A	0	0	m.1494C>T^d^	9	0.06
c.92+5G>C	0	0	***MT‐RNR1*** Total	72	0.52
c.94delC	0	0	**Hearing loss Total** ^e^	**678**	**4.87**
***HBB*** Total	477	3.43			
**Thalassemia** Total^b^	**1228**	**8.82**	**Total** ^f^	**2109**	**15.14**

*Note:* The RefSeq IDs of *SMN1, HBA1, HBA2, HBB, GJB2, SLC26A4, GJB3*, and *MT‐RNR1* are NM_000344.4, NM_000558.5, NM_000517.6, NM_000518.5, NM_004004.6, NM_000441.2, NM_024009.3, and NC_012920.1, respectively.

^a^ Among these, 11 cases carried two different variants of *HBA1/HBA2*.

^b^ Among these, 25 cases carried both variants of *HBA1/HBA2* and *HBB*.

^c^ Heteroplasmic sequence variants found in seven cases and homoplasmic sequence variants found in 56 cases.

^d^ Heteroplasmic sequence variants found in eight cases and homoplasmic sequence variants found in one case.

^e^ Among these, 10 cases carried variants of different deafness susceptibility genes.

^f^ Three cases carried common variants in genes for all three diseases (SMA, HL, and thalassemia), 85 carried common variants in genes related to two of these diseases, and 2021 carried variants in genes related to one of these diseases.

**TABLE 2:** Homozygous variants and compound heterozygous variants identified in carrier screening.

Gene	Variant	Inheritance	Cases	Clinical features
*GJB3*	c.538C>T	Homozygous	1	28 years old, passed the hearing test, with no abnormalities found in follow‐up audiological assessments.
*SMN1*	Exon7 deletion	Homozygous	1	25 years old, no clinical phenotypes associated with Spinal Muscular Atrophy (SMA), serum creatine kinase level of 73 U/L (normal range), and no significant abnormalities noted on electromyography (EMG).
*HBA1/HBA2*	‐α^3.7^	Homozygous	8	Apart from mild anemia in some individuals, with hemoglobin levels ranging from 109 to 123 g/L, there were no other clinical phenotypes observed.
*HBA1/HBA2*	‐α^3.7^; ‐α^4.2^	Compound heterozygous	4	
*HBA1/HBA2*	‐α^3.7^; *HBA2*:c.377T>C	Compound heterozygous	1	
*HBA1/HBA2*	‐α^3.7^; *HBA2*:c.427T>C	Compound heterozygous	1	
*HBA2*	‐α^4.2^; c.427T>C	Compound heterozygous	1	
*HBA2*	‐α^4.2^; c.377T>C	Compound heterozygous	1	

*Note:* The RefSeq IDs of *GJB3*, *SMN1*, *HBA1*, and *HBA2* are NM_024009.3, NM_000344.4, NM_000558.5, and NM_000517.6, respectively.

In addition, the number of cases with deletion of *SMN1* exon 8 was counted, although cases without deletion of exon 7 were not included in the positive carrier cases. A total of 347 cases were found to have deletion of exon 8: 80 had heterozygous deletion of exon 8 without deletion of exon 7, 265 had heterozygous deletion of exon 8 and exon 7, one had heterozygous deletion of exon 8 with homozygous deletion of exon 7 (*SMN2* three copies), and one had homozygous deletion of exon 8 with heterozygous deletion of exon 7 (*SMN2* two copies).

### 3.2 Genetic Screening of Participating Husbands

Recall of the 1978 husbands of the female carriers was attempted (the 131 cases that were found to have variants only in *MT‐RNR1* and/or *GJB3* were excluded). Finally, 1117 husbands (1117/1978, 56.47%) were successfully recalled. Among them, 1050 were tested for a single gene, 65 were tested for two genes, and two were tested for three genes. The abnormal variant rates of *SMN1*, *GJB2*, *SLC26A4*, *HBA1/HBA2*, and *HBB* were 2.67% (5/187), 22.85% (61/267), 7% (7/100), 6.94% (30/432), and 4% (8/200), respectively. A total of 111 (111/13926; 0.80%) at‐risk couples were found to carry variants in the same gene (Table 3).

**TABLE 3:** Variants of the same gene detected in 111 couples.

Gene	Women of reproductive age			Husbands			Cases
	Variation	Inheritance	Classification	Variation	Inheritance	Classification	
*GJB2*	c.176_191del	het	P	c.109G>A	het	P	5
*GJB2*	c.235delC	het	P	c.109G>A	het	P	35
*GJB2*	c.235delC	het	P	c.88A>G	het	VUS	1
*GJB2*	c.235delC	het	P	c.583A>G	het	P	1
*GJB2*	c.235delC	het	P	c.235delC	het	P	4
*GJB2*	c.235delC	het	P	c.478G>A	het	VUS	1
*GJB2*	c.235delC	het	P	c.299_300delAT	het	P	1
*GJB2*	c.235delC	het	P	c.571T>C	het	VUS	1
*GJB2*	c.235delC	het	P	c.176_191del	het	P	1
*GJB2*	c.235delC	het	P	c.109G>A	hom	P	2
*GJB2*	c.299_300delAT	het	P	c.235delC	het	P	2
*GJB2*	c.299_300delAT	het	P	c.187G>T	het	VUS	1
*GJB2*	c.299_300delAT	het	P	c.109G>A	het	P	2
*GJB2*	c.508_511dupAACG	het	P	c.109G>A	het	P	3
*GJB2*	c.508_511dupAACG	het	P	c.109G>A	hom	P	1
*SLC26A4*	c.2168A>G	het	P	c.2233delA	het	VUS	1
*SLC26A4*	c.2168A>G	het	P	c.1174A>T	het	P	1
*SLC26A4*	c.1174A>T	het	P	c.1829C>A	het	P	1
*SLC26A4*	c.919‐2A>G	het	P	c.919‐2A>G	het	P	3
*SLC26A4*	c.919‐2A>G	het	P	c.1181_1183delTCT	het	P	1
*HBA1/HBA2*	‐α^3.7^	het	P	‐α^3.7^	het	P	4
*HBA1/HBA2*	‐α^3.7^	het	P	‐^SEA^	het	P	5
*HBA1/HBA2*	‐α^3.7^	het	P	‐α^4.2^	het	P	1
*HBA1/HBA2*	‐^SEA^	het	P	‐^SEA^	het	P	7
*HBA1/HBA2*	‐^SEA^	het	P	‐α^3.7^	het	P	5
*HBA1/HBA2*	‐^SEA^	het	P	‐α^4.2^	het	P	2
*HBA1/HBA2*	‐^SEA^	het	P	*HBA2*:c.427T>C	het	P	1
*HBA1/HBA2*	‐α^4.2^	het	P	‐α^3.7^	het	P	3
*HBA1/HBA2*	*HBA2*:c.427T>C	het	P	‐α^3.7^	het	P	2
*HBB*	c.52A>T	het	P	c.126_129delCTTT	het	P	2
*HBB*	c.316‐197C>T	het	P	c.79G>A	het	P	1
*HBB*	c.316‐197C>T	het	P	c.316‐197C>T	het	P	1
*HBB*	c.316‐197C>T	het	P	c.‐78A>G	het	P	1
*HBB*	c.52A>T	het	P	c.52A>T	het	P	1
*HBB*	c.126_129delCTTT	het	P	c.52A>T	het	P	2
*SMN1*	Exon7 deletion	het	P	Exon7 deletion	het	P	5

*Note:* The RefSeq IDs of *GJB2*, *SLC26A4*, *HBA1*, *HBA2*, *HBB*, and *SMN1* are NM_004004.6, NM_000441.2, NM_000558.5, NM_000517.6, NM_000518.5, and NM_000344.4, respectively.

Abbreviations: het, heterozygous; P, pathogenic; VUS, variant of uncertain significance.

### 3.3 Prenatal Diagnosis Results for High‐Risk Families

**FIGURE 2:**
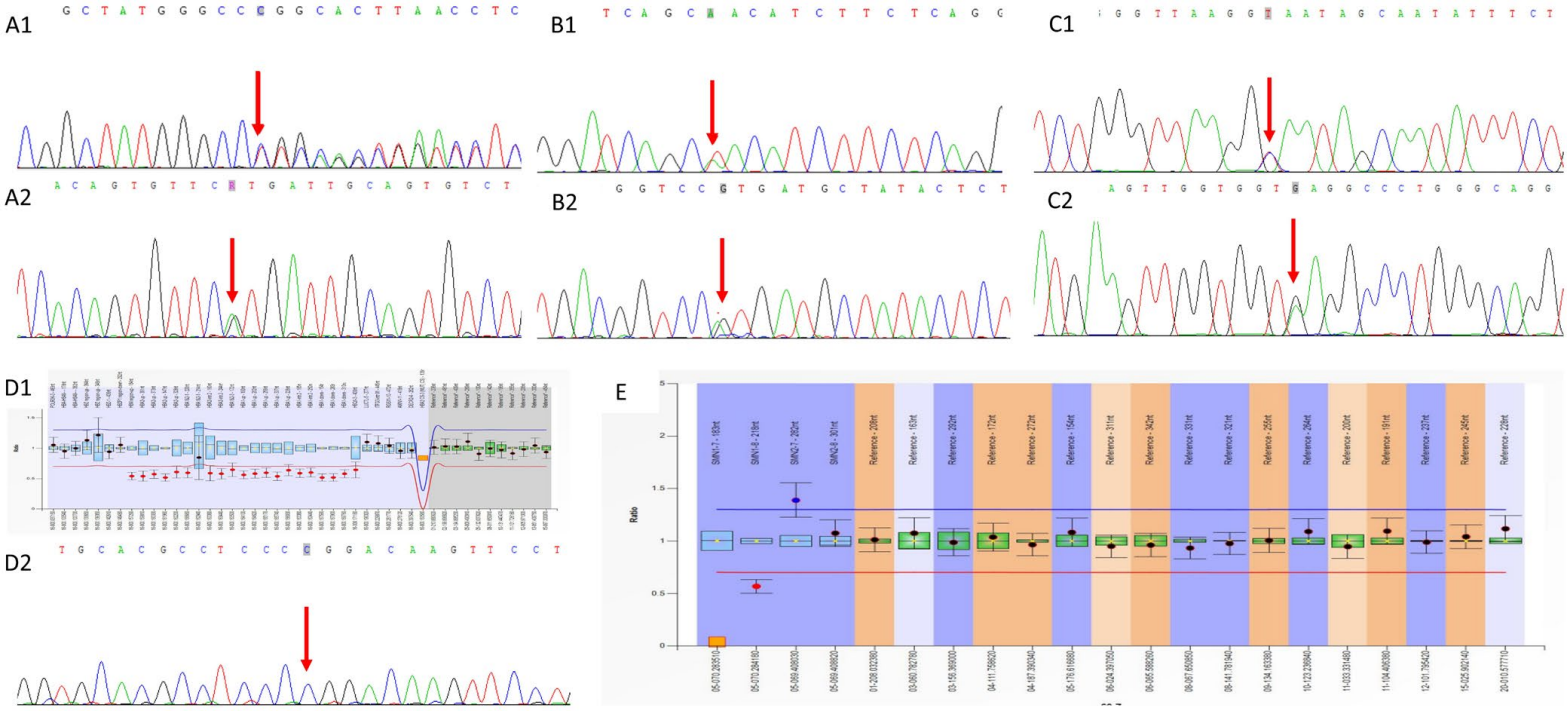
Sanger sequencing or MLPA results of five prenatally diagnosed families. (A) Compound heterozygous for c.235delC (A1) and c.583A>G (A2) in *GJB2* (NM_004004.6) of family 14; (B) Compound heterozygous for c.1174A>T (B1) and c.2168A>G (B2) in *SLC26A4* (NM_000441.2) of family 15; (C) Compound heterozygous for c.316‐197C>T (C1) and c.79G>A (C2) in *HBB* (NM_000518.5) of family 42; (D) Compound heterozygous for ‐^SEA^ (D1) and *HBA2*:c.427T>C (D2) in *HBA1* (NM_000558.5) and *HBA2* (NM_000517.6) of family 36; (E) Homozygous deletion of *SMN1* (NM_000344.4) exon 7 of family 43.

After genetic counseling, 47 high‐risk families decided to undergo prenatal diagnosis in our hospital, including 14 couples with *GJB2* variants, 16 couples with *HBA1/HBA2* variants, six couples with *HBB* variants, six couples with *SLC26A4* variants, and five couples with *SMN1* exon 7 deletion. The results of the peripheral blood samples from the 47 couples were consistent with the results of the saliva samples. Prenatal diagnosis revealed that 11 fetuses were wild type, 13 were HL or SMA carriers, one had a risk of HL with homozygous *GJB2*:c.109G>A, one was diagnosed with SMA, six were diagnosed with HL, nine were diagnosed with silent or minor thalassemia, and six were diagnosed with intermedia or major thalassemia. Overall, there were 13 (13/13926; 0.93‰) affected pregnancies. Ultimately, these 13 families chose to terminate the pregnancies; one fetus which was diagnosed as a carrier of HL also had trisomy 21. See Table 4 and Figure 2.

**TABLE 4:** Prenatal diagnosis of 47 high‐risk families.

Family	Gene	Pathogenic variants (inheritance)			Sample type of fetus	Status of fetus	Pregnancy outcome
		Pregnant women	Husbands	Fetus			
1	*GJB2*	c.235delC (het)	c.109G>A (het)	c.109G>A (het)	Amniotic fluid	Normal carrier	Born alive
2	*GJB2*	c.235delC (het)	c.109G>A (het)	c.235delC (het)	Amniotic fluid	Normal carrier	Born alive
3	*GJB2*	c.235delC (het)	c.109G>A (het)	c.109G>A (het)	Amniotic fluid	Normal carrier	Born alive
4	*GJB2*	c.235delC (het); c.109G>A^a^ (het)	c.109G>A (het)	Wild type	Amniotic fluid	Normal	Born alive
5	*GJB2*	c.299_300delAT (het)	c.109G>A (het)	c.109G>A (het)	Amniotic fluid	Normal carrier	Born alive
6	*GJB2*	c.508_511dupAACG (het)	c.109G>A (het)	Wild type	Amniotic fluid	Normal	Born alive
7	*GJB2*	c.235delC; c.109G>A^a^ (com‐het)	c.109G>A (hom)	c.109G>A (hom)	Amniotic fluid	Possible patient (hearing loss)	Born alive
8	*GJB2*	c.235delC (het)	c.176_191del (het)	c.235delC (het)	Amniotic fluid	Normal carrier	Born alive
9	*GJB2*	c.235delC (het)	c.235delC (het)	c.235delC (hom)	Amniotic fluid	Patient (hearing loss)	TOP
10	*GJB2*	c.235delC (het)	c.235delC (het)	c.235delC (hom)	Amniotic fluid	Patient (hearing loss)	TOP
11	*GJB2*	c.235delC (het)	c.235delC (het)	c.235delC (het)	Amniotic fluid	Normal carrier	Born alive
12	*GJB2*	c.235delC (het)	c.235delC (het)	c.235delC (het)	Amniotic fluid	Normal carrier	TOP^b^
13	*GJB2*	c.235delC (het)	c.299_300delAT (het)	c.235delC; c.299_300delAT (com‐het)	Amniotic fluid	Patient (hearing loss)	TOP
14	*GJB2*	c.235delC (het)	c.583A>G (het)	c.235delC; c.583A>G (com‐het)	Amniotic fluid	Patient (hearing loss)	TOP
15	*SLC26A4*	c.2168A>G (het)	c.1174A>T (het)	c.1174A>T; c.2168A>G (com‐het)	Amniotic fluid	Patient (hearing loss)	TOP
16	*SLC26A4*	c.919‐2A>G (het)	c.1181_1183delTCT (het)	Wild type	Amniotic fluid	Normal	Born alive
17	*SLC26A4*	c.1174A>T (het)	c.1829C>A (het)	c.1174A>T (het)	Amniotic fluid	Normal carrier	Born alive
18	*SLC26A4*	c.919‐2A>G (het)	c.919‐2A>G (het)	c.919‐2A>G (het)	Amniotic fluid	Normal carrier	Born alive
19	*SLC26A4*	c.919‐2A>G (het)	c.919‐2A>G (het)	c.919‐2A>G (hom)	Amniotic fluid	Patient (hearing loss)	TOP
20	*SLC26A4*	c.919‐2A>G (het)	c.919‐2A>G (het)	c.919‐2A>G (het)	Amniotic fluid	Normal carrier	Born alive
21	*HBA1/HBA2*	‐^SEA^ (het)	‐^SEA^ (het)	‐^SEA^ (het)	Amniotic fluid	Minor carrier (α‐thalassaemia)	Born alive
22	*HBA1/HBA2*	‐^SEA^ (het)	‐^SEA^ (het)	Wild type	Amniotic fluid	Normal	Born alive
23	*HBA1/HBA2*	‐^SEA^ (het)	‐^SEA^ (het)	‐^SEA^ (het)	Amniotic fluid	Minor carrier (α‐thalassaemia)	Born alive
24	*HBA1/HBA2*	‐^SEA^ (het)	‐^SEA^ (het)	‐^SEA^ (hom)	Amniotic fluid	Patient (Hb Bart's)	TOP
25	*HBA1/HBA2*	‐^SEA^ (het)	‐^SEA^ (het)	‐^SEA^ (het)	Amniotic fluid	Minor carrier (α‐thalassemia)	Born alive
26	*HBA1/HBA2*	‐^SEA^ (het)	‐^SEA^ (het)	Wild type	Amniotic fluid	Normal	Born alive
27	*HBA1/HBA2*	‐^SEA^ (het)	‐^SEA^ (het)	‐^SEA^ (het)	Amniotic fluid	Minor carrier (α‐thalassemia)	Born alive
28	*HBA1/HBA2*	‐α^3.7^ (het)	‐^SEA^ (het)	Wild type	Amniotic fluid	Normal	Born alive
29	*HBA1/HBA2*	‐α^3.7^ (het)	‐^SEA^ (het)	‐^SEA^; ‐α^3.7^ (com‐het)	Amniotic fluid	Patient (Hb H)	TOP
30	*HBA1/HBA2*	‐α^3.7^ (het)	‐^SEA^ (het)	‐α^3.7^ (het)	Amniotic fluid	Silent carrier (α‐thalassemia)	Born alive
31	*HBA1/HBA2*	‐α^3.7^ (het)	‐^SEA^ (het)	‐α^3.7^ (het)	Amniotic fluid	Silent carrier (α‐thalassemia)	Born alive
32	*HBA1/HBA2*	‐^SEA^ (het)	‐α^3.7^ (het)	‐^SEA^; ‐α^3.7^ (com‐het)	Amniotic fluid	Patient (Hb H)	born alive
33	*HBA1/HBA2*	‐^SEA^ (het)	‐α^3.7^ (het)	‐^SEA^ (het)	Amniotic fluid	Minor carrier (α‐thalassemia)	Born alive
34	*HBA1/HBA2*	‐^SEA^ (het)	‐α^3.7^ (het)	Wild type	Amniotic fluid	Normal	Born alive
35	*HBA1/HBA2*	‐^SEA^ (het)	‐α^4.2^ (het)	‐^SEA^; ‐α^4.2^ (com‐het)	Amniotic fluid	Patient (Hb H)	TOP
36	*HBA1/HBA2*	‐^SEA^ (het)	*HBA2*:c.427T>C (het)	‐^SEA^; *HBA2*:c.427T>C (com‐het)	Amniotic fluid	Patient (Hb H)	TOP
37	*HBB*	c.316‐197C>T (het)	c.‐78A>G (het)	Wild type	Amniotic fluid	Normal	Born alive
38	*HBB*	c.126_129delCTTT (het)	c.52A>T (het)	Wild type	Amniotic fluid	Normal	Born alive
39	*HBB*	c.126_129delCTTT (het)	c.52A>T (het)	Wild type	Amniotic fluid	Normal	Born alive
40	*HBB*	c.52A>T (het)	c.126_129delCTTT (het)	c.126_129delCTTT (het)	Amniotic fluid	Minor carrier (β‐thalassemia)	Born alive
41	*HBB*	c.316‐197C>T (het)	c.316‐197C>T (het)	c.316‐197C>T (het)	Amniotic fluid	Minor carrier (β‐thalassemia)	Born alive
42	*HBB*	c.316‐197C>T (het)	c.79G>A (het)	c.316‐197C>T; c.79G>A (com‐het)	Amniotic fluid	Patient (β‐thalassemia major/intermedia)	TOP
43	*SMN1*	Exon7 deletion (het)	Exon7 deletion (het)	Exon7 deletion (hom)	Amniotic fluid	Patient (SMA)	TOP
44	*SMN1*	Exon7 deletion (het)	Exon7 deletion (het)	Exon7 deletion (het)	Chorionic villus	Normal carrier	Born alive
45	*SMN1*	Exon7 deletion (het)	Exon7 deletion (het)	Exon7 deletion (het)	Amniotic fluid	Normal carrier	Born alive
46	*SMN1*	Exon7 deletion (het)	Exon7 deletion (het)	Wild type	Amniotic fluid	Normal	Born alive
47	*SMN1*	Exon7 deletion (het)	Exon7 deletion (het)	Exon7 deletion (het)	Amniotic fluid	Normal carrier	Born alive

*Note:* The RefSeq IDs of *GJB2*, *SLC26A4*, *HBA1*, *HBA2*, *HBB*, and *SMN1* are NM_004004.6, NM_000441.2, NM_000558.5, NM_000517.6, NM_000518.5, and NM_000344.4, respectively.

Abbreviations: com‐het, compound heterozygous; Hb, hemoglobin; het, heterozygous; hom, homozygous; SMA, spinal muscular atrophy; TOP, termination of pregnancy.

^a^ The variant was detected by subsequent Sanger sequencing.

^b^ Trisomy 21 was also detected in this fetus.

## 4 Discussion

According to previous reports, although SMA, hereditary HL, and thalassemia are common monogenic genetic diseases, the carrier frequencies of common variants in genes for these diseases show regional differences (Sugarman et al. 2012; Yin et al. 2014; Zhang et al. 2023). This study found that the carrier frequencies of SMA, HL, and thalassemia in women of reproductive age in Southern Zhejiang were 2.11%, 4.87%, and 8.82%, respectively. The most common variants in HL carriers were c.235delC of *GJB2*, c.919‐2A>G of *SLC26A4*, and m.1555A>G of *MT‐RNR1*, and those in thalassemia carriers were ‐^SEA^, ‐α^3.7^, and *HBB*:c.126_129delCTTT. The local carrier frequency of SMA in this study is higher than those reported for most regions in China (Zhou et al. 2024; Zhang et al. 2020), and the frequency of thalassemia is lower than the estimates reported for most of Southeastern China, but higher than that of Fujian Province (6.8%; Huang et al. 2019). Compared with a recent HL carrier study of 3,555,336 Chinese newborns (Zhang et al. 2023), the local carrier frequency of *SLC26A4* in this study is lower (1.16% vs. 2.05%), while there are slightly higher frequencies of *GJB2, GJB3*, and *MT‐RNR1* in this study (2.72% vs. 2.53%, 0.54% vs. 0.37%, and 0.52% vs. 0.25%, respectively). These findings confirm that SMA, HL, and thalassemia are highly prevalent in Southern Zhejiang and show some regional specificity, as compared with large population‐based surveys in China in recent years. In general, the carrier frequency among the male participating spouses was higher than that among the female subjects, with the largest difference being the inclusion of the variant c.109G>A of G*JB2* in the former. The prevalence of this variant (17.98%, 48/267) was much higher than previously reported (Liang et al. 2023; Liu et al. 2019). A study with a larger sample size should be performed to verify this finding.

Homozygous deletion of *SMN1* exon 7 is the main cause of SMA while *SMN1* exon 8 is in the noncoding region and its correlation with SMA has not been established. While there are currently some reports of SMA patients with homozygous deletion of *SMN1* exon 8 (Behera and Kumar 2020; Maiti, Bhattacharya, and Yadav 2012), suggesting that exon 8 is associated with SMA, we identified a 30‐year‐old unaffected female carrying homozygous deletion of *SMN1* exon 8, which does not support this notion. Additionally, another 25‐year‐old unaffected female with homozygous deletion of *SMN1* exon 7 was identified in our study, and subsequent MLPA testing revealed three copies of *SMN2*. It is known that probands with an *SMN2* copy number ≥ 3 potentially have a milder phenotype, but adult‐onset patients with *SMN2* 3 copies are still very rare (Wirth et al. 2020).

The provision of genetic counseling and prenatal diagnosis for high‐risk families requires particular prudence. In the current study, affected families that met the following criteria were excluded from prenatal diagnosis. First, prenatal diagnosis was not performed for families in which the female was a carrier, but the male spouse only had a VUS variant. Among the nine additional variants of *GJB2* and *SLC26A4* detected in the male spouses, aside from c.109G>A (PS4+PP1_Strong) and c.583A>G (PM2+PM3_Verystrong+PP3) of *GJB2*, and c.1181_1183delTCT (PM3_Strong+PM4+PM2+PP1+PP4) and c.1829C>A (PVS1+PM3+PM2_Supporting+PM3_Supporting) of *SLC26A4*, which are classified as pathogenic, c.88A>G, c.187G>T, c.478G>A, and c.571T>C of *GJB2* and c.2233delA of *SLC26A4* are classified as variants of uncertain significance, even though these four variants of uncertain significance of *GJB2* have been reported in HL patients (Chai et al. 2014; Roux et al. 2004; Xu et al. 2002; Zheng et al. 2015). Second, homoplasmic or heteroplasmic variations of *MT‐RNR1* detected in the female subjects were excluded from prenatal diagnosis. There is no doubt that these variants are inherited maternally, so the screening results are more helpful in explaining the risks of aminoglycoside use. Third, variations of *GJB3* detected in the female subjects were excluded from prenatal diagnosis. Previous reports have suggested that c.538C>T and c.547G>A of *GJB3* are responsible for autosomal‐dominant non‐syndromic sensorineural hearing impairment; however, these suggestions have been contested by researchers in recent years (Huang et al. 2017). We detected these two variants in 75 unaffected female subjects, and one of them, who had homozygous c.538C>T, passed the hearing test. In addition, the choice of pregnant women should be fully considered when offering genetic counseling to families where the husband only carries c.109G>A of *GJB2* or the offspring have, at most, intermedia thalassemia. Although c.109G>A is a pathogenic variant according to the ACMG, confirmation of the clinical phenotype of the variant through prenatal diagnosis is still difficult because of the heterogeneity in the hearing phenotype resulting from c.109G>A. The variant presents with low penetrance, mild to profound sensorineural HL, and can cause delayed and progressive deafness (Lin et al. 2022).

Saliva samples have similar DNA contents to blood samples (Siuta et al. 2023), and evidence suggests that DNA from saliva samples, collected using standard procedures, can be successfully used for the sequencing of exomes and full genomes (Kidd et al. 2014). Saliva is advantageous as it is widely applicable and non‐invasive; however, there are limited reports of saliva sample‐based genomic sequencing (Botos, Szatmári, and Nagy 2023; Feliciano et al. 2019). Advances in genomic testing are leading to the revolutionary upheaval of carrier screening. As large‐scale carrier screening projects emerge, challenges have surfaced, including insufficient population awareness, the high cost of extended carrier screening, insufficient resources in primary hospitals, a limited genetic counseling ability of primary doctors, and the high mobility of the population. Therefore, there is an urgent need for an efficient and timely screening mode for important variants that can be widely applied. Our saliva‐based non‐invasive carrier screening for common variants can effectively alleviate some of the above problems. In this study, 47 high‐risk families underwent prenatal diagnosis, and a total of 13 affected pregnancies were identified. Due to the insufficient recall rate of male spouses, the actual incidence of affected pregnancies is likely higher. These findings indicate that this screening method is highly feasible in this region.

Our study has several shortcomings that should be noted. First, we have 112 screening sites in our region, and some doctors have received insufficient genetic counseling training; therefore, some subjects with a mild thalassemia phenotype were included, which led to a slightly higher positive rate of thalassemia. Second, the standardization of sampling needs to be strengthened. About 0.1% of the samples were recollected because the DNA content did not meet the detection standard (300 ng). Third, while the female participants underwent screening for common variants, they could have also been carrying rare variants that were not investigated in this study. Therefore, there remains a residual risk for individuals or couples who obtained negative results in the current study. Finally, the husband recall rate was not high. This may be due to a limited awareness of genetics among the population. Thus, the genetic knowledge of the population still needs to be strengthened.

## 5 Conclusion

In summary, our study established, for the first time, a saliva‐based non‐invasive screening mode for SMA, HL, and thalassemia. Through the large‐scale application of this effective and comprehensive carrier screening method, the carrier frequencies of SMA, deafness, and thalassemia in women of reproductive age from South Zhejiang were determined, and variants with high incidence were initially screened, providing references for carrier screening in this region. At the same time, genetic counseling and fertility guidance were conducted, effectively increasing reproductive autonomy for at‐risk couples.

## Author Contributions

All authors contributed to the study conception and design. Chenyang Xu and Xueqin Xu designed the study, interpreted the clinical data, and wrote the article. Yanbao Xiang, Huanzheng Li, Yunzhi Xu, Qifan Ma, and Xiaoling Lin collected samples, genotyped the cases, and finished the follow‐up. Xueqin Xu and Shaohua Tang performed genetic counseling. Chenyang Xu and Yanbao Xiang helped in statistical analysis. All authors read and approved the final manuscript.

## Ethics Statement

This study has been approved by the corresponding authors' Institutional Review Board.

## Consent

Informed consent has been obtained from all individuals included in this study.

## Conflicts of Interest

The authors declare no conflicts of interest.

## Data Availability

The data providing the evidence of the study is available from the corresponding author upon reasonable request.
